# Highly Conductive Nanocrystalline Diamond Films and Electronic Metallization Scheme

**DOI:** 10.3390/ma14164484

**Published:** 2021-08-10

**Authors:** Xin Chen, Markus Mohr, Kai Brühne, Hans-Jörg Fecht

**Affiliations:** Institute of Functional Nanosystems, Ulm University, 89081 Ulm, Germany; markus.mohr@uni-ulm.de (M.M.); kai.bruehne@uni-ulm.de (K.B.); hans.fecht@uni-ulm.de (H.-J.F.)

**Keywords:** nanocrystalline diamond, hot-filament CVD, contact resistance, reactive-ion etching

## Abstract

By using a methane and hydrogen process gas mixture in an appropriate hot-filament CVD process without further dopant, high electrical conductivity of over 100 S/cm has been achieved in nanocrystalline diamond films deposited on silicon single-crystalline substrates. Furthermore, it was found that an oxygen reactive-ion etching process (O-RIE) can improve the diamond film surface’s electron affinity, thus reducing the specific contact resistance. The reduction of the specific contact resistance by a factor of up to 16 was realized by the oxygen ion etching process, down to $6\times 10^{-6}{\;\Omega \mathrm{cm}}^{2}$. We provide a qualitative explanation for the mechanism behind the contact resistance reduction in terms of the electron affinity of the diamond surface. With the aid of XPS, AFM, and surface wetting measurements, we confirmed that a higher surface electron affinity is responsible for the lower specific contact resistance of the oxygen-terminated nanocrystalline diamond films.

## 1. Introduction

Diamond has outstanding mechanical properties, together with chemical inertness and high thermal conductivity [1]. The success of different chemical vapor deposition (CVD) techniques for synthesizing diamond has greatly expanded its laboratory study and industrial application [2]. For example, the hot-filament chemical vapor deposition (HFCVD) technique is widely used for deposition on three-dimensional objects and can easily be scaled to large coating areas [1]. The microwave plasma chemical vapor deposition (MPCVD) technique is more suitable for coating of optical products, thanks to its low contamination from electrodes [3].

By tailoring the electronic structure through chemical doping or nanostructure formation, it becomes an attractive material for electronic devices and sensors. Examples include high-voltage diamond diodes with intrinsic and boron-doped layers [4], diamond power devices with delta-doped FETs [5], piezoresistive sensors [6], hybrid silicon-nanorystalline diamond membrane pressure sensors [7], and nanodiamond actors for thermally generated cavitation [8], based on conductive n-type (ultra-)nanocrystalline diamond (UNCD). Despite impressive figures of merit on theoretical electrical device performance, the diamond-based electron device technology is limited by the difficulties related with diamond doping [9] or their various surface (ohmic or rectifying) contacts’ fabrication [10].

Due to the extremely strong covalent bonding of carbon atoms (sp^3^ bonds) in diamond crystals, dopants have a high ionization energy, resulting in low activation at room temperature [1]. Diamond films with ultra-nano grain size can also exhibit n-type electrical conductivity due to their unique grain boundary structure [11,12,13]. On the other hand, several simulations have revealed that, in diamond, part of the excess energy of the grain boundary is relaxed by changing the hybridization of the carbon atoms [14,15]. This leads to re-bonding into more sp^2^ bonded carbon structures and disordered grain boundaries [14,15]. That in turn enhances the electrical conductivity when the diamond grain size is in the nanometer scale [13].

Furthermore, ohmic contacts with low contact resistance are difficult to form to diamond due to its wide bandgap and its interface barrier height with contacts [16,17]. Effective ohmic contacts were obtained only by forming a high concentration surface layer via in-situ doping or by a suitable annealing treatment [10]. Nevertheless, earlier theoretical [18] and experimental [19,20] attempts showed that surface states were an unavoidable result of the termination of their lattice, hence affecting the interface barrier and resistance [16].

A high conductivity with reduced ohmic contact resistance allows devices on diamond films to perform with improved sensitivity and energy efficiency. This opens the way for the development of new devices based on nanoelectronics. For instance, reducing the contact resistance between metal contacts and piezoresistive UNCD sensors enables an increased sensor sensitivity [6,7]. The low contact resistance also means less power loss for thermal cavitation actuators based on highly conductive diamond films [8]. Moreover, this combination was also working as an electrode substrate in a biosensor [21] and will be used in the future as a practical multilayer capacitor for a wear sensor, consisting of sequential non-conductive and conductive diamond film layers.

In this paper, we present an investigation of the synthesis of non-conductive and conductive nanocrystalline undoped diamond films onto Si wafers with controllable specific electrical conductivity ($\sigma_{f},\;S/\mathrm{cm}$) in the range of $9.86\times 10^{-6}$ to 133.45 S/cm, and additionally, we show how to minimize the specific contact resistance ($\rho_{c},{\;\Omega \mathrm{cm}}^{2}$) between conductive diamond films and different metals, using different metallization and oxygen ion etching processes.

With an increase of the methane (${\mathrm{CH}}_{4}$) composition in the CH_4_/H_2_ precursor gas mixture, a strong increase in the diamond films’ specific electrical conductivity occurred. On the other hand, the specific contact resistance of diamond films was reduced by a factor of 5–16, by an optimized contact material selection and after an oxygen reactive-ion etching (O-RIE) process on the film’s surface.

## 2. Materials and Methods

Nanocrystalline diamond films (NCD) and electrically high-conductive (ultra-)nanocrystalline diamond films (UNCD) were deposited onto 4” Si wafers with a controlled thickness of typically 1 µm, using the HFCVD process, after the Si wafers were seeded by ultrasonication inside a nanocrystalline diamond solution, which resulted in a seeding density of $10^{11}{\;\mathrm{cm}}^{-2}$ and an average seed size of 3–5 nm.

Tungsten was used as filament material, and the filaments were resistively heated to around 2000 °C. Methane (${\mathrm{CH}}_{4}$), together with hydrogen ($H_{2}$), was used as a process gas mixture. The ratio of the methane to hydrogen gas flow was varied between 3%, 4%, 5%, and 6%, corresponding to samples I, II, III, and IV.

Before the seeding and deposition process, the Si wafers’ surfaces were oxidized by heating them in a furnace under atmospheric conditions in order to build up an insulating ${\mathrm{SiO}}_{2}$ layer (~100–200 nm thickness) and to prevent the influence of the Si wafer’s electrical conductivity on the measurement of the diamond films’ electrical conductivity.

The surface morphology and roughness of diamond films were captured and measured by SEM (scanning electron microscopy) and AFM (atomic force microscopy), respectively. X-ray diffraction (XRD) measurements in a standard Bragg–Brentano geometry using a Cu Kα X-ray source were used, on the one hand to prove the existence of crystalline diamond grains in the film, and on the other hand, for approximating the diamond film’s average grain size, using Scherrer’s equation and considering the FWHM (full width at half maximum) of the diamond (111)-peak by 2θ=43.9°.

Raman measurements were obtained using a diode-pumped solid-state laser (DPSSL) with a wavelength of 532 nm, as Raman spectroscopy is highly sensitive to amorphous and graphitic carbon materials due to the resonance of laser excitation energy, so as to obtain information about the bonding structural properties of the grain boundary region.

**Figure 1:**
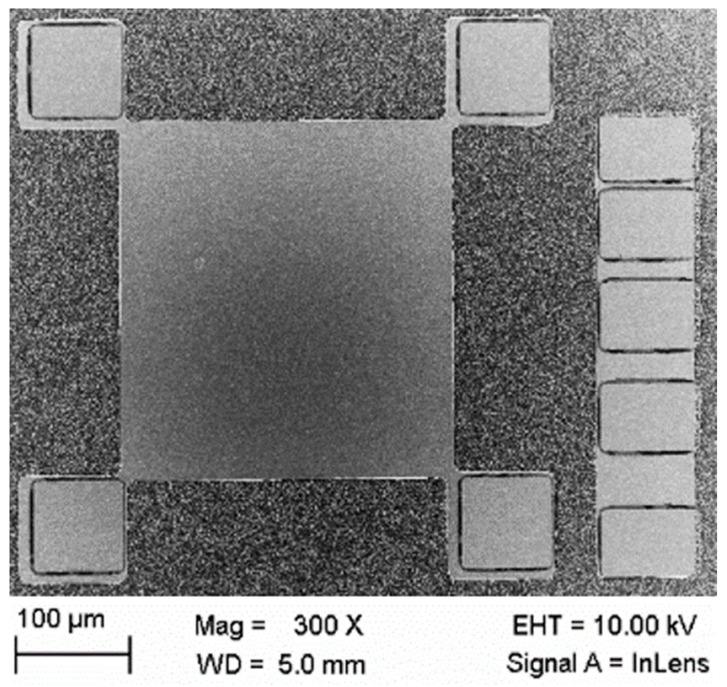
VDP (left) and LTLM (right) micro-structures prepared photolithographically.

Microstructures for electric characterization were fabricated by micro-technology methods. In order to pattern the conductive nanocrystalline diamond layer, a structured aluminum mask was deposited in a lift-off process by means of photolithography and thermal evaporation. The patterning of the UNCD layers was performed by an oxygen plasma etching process. Besides 4-point-measurements, Van-der-Pauw (VDP, 300 µm × 320 µm inside 4 contact pads) and Linear-Transmission-Line-Methods (LTLM, contact pads distance, respectively, 10 µm, 20 µm, 30 µm, and 50 µm from $l_{1}$ to $l_{4}$) micro-structures were prepared by a photolithography process in a clean room (Figure 1), for measurement and evaluation of the specific electrical conductivity of NCD and UNCD. In addition, LTLM structures were used, especially for those two UNCD samples (specific electrical conductivity by 12.93 S/cm, Sample Ⅲ), whose surfaces were pre-treated with an oxygen ion etching process with different plasma power values.

In the micro-technology of electronic diamond devices, gold is preferentially used as a main part of the metallization, when connecting active UNCD elements or when building contact pads, due to its chemical inertness and good electrical conductivity. However, gold is not a carbide-forming element and hence does not present a good mechanical bond with and adhesion to diamond. Gold was nonetheless used as contact metal, together with the carbide-forming metals tantalum (Ta) and molybdenum (Mo) for the UNCD film. The oxidation of the two carbide-forming contact materials was prevented by the subsequent coating with a thin Au layer. This layer was sputtered onto the contact material in the same magnetron sputtering device, without exposure of the sample to air in between. Since Ta showed a generally smaller specific contact resistance, it was chosen for further experiments where the UNCD film surface was pre-treated with different O-RIE processes. Table 1 shows an overview of the samples for the contact resistance measurements. Furthermore, we performed XPS (X-ray photoelectron spectroscopy, using Al-Kα irradiation source, 1486 eV) measurements on the diamond films’ surfaces to gain insights into the reasons for a reduced contact resistance after O-RIE.

## 3. Results and Discussion

**Figure 2:**
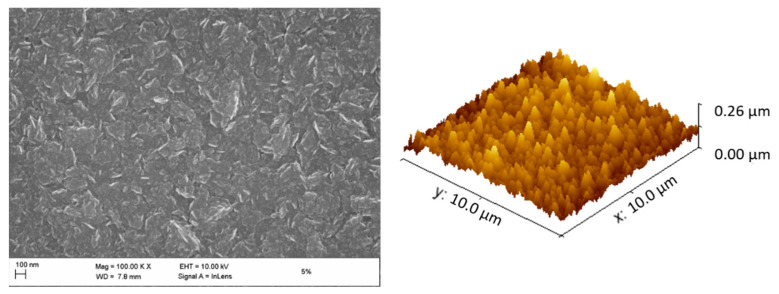
SEM and AFM images of diamond film sample Ⅲ with a methane ratio of 5% in the process gas.

**Figure 3:**
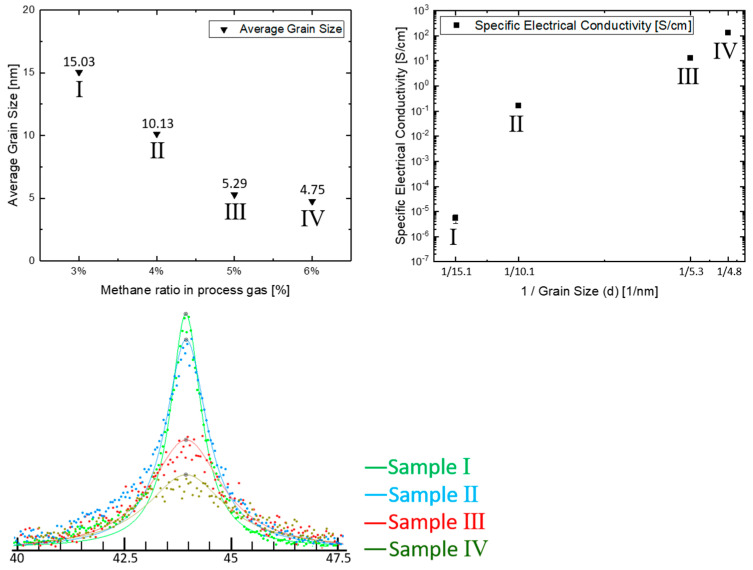
Evaluation of average grain size (**top-left**), specific electrical conductivity (**top right**, evaluated with “4 points measurement”), and XRD-patterns (**bottom**) of four diamond film samples.

Taking sample Ⅲ as an example, both its SEM and AFM images (Figure 2) show a smooth and homogeneous surface morphology. The SEM image indicates that the film consisted of small diamond grains with an average size below 20 nm. This is further confirmed by XRD (Figure 3, top-left and bottom). The average grain size of the diamond films decreased from about 15 nm (sample Ⅰ) to around 5 nm (sample Ⅳ), when the methane ratio increased from 3% to 6%. The average surface roughness ($R_{\mathrm{rms}}$, root mean square roughness) obtained from the AFM measurements was in a similar range for all four samples. The roughness ranged from 20–35 nm, since all those samples (Ⅰ, Ⅱ, Ⅲ, Ⅳ) consisted of nanograins smaller than 20 nm, resulting from a growth process with a significant re-nucleation rate [22]. Together with the reduction of their grain size, the specific electrical conductivity of those four samples drastically increased from $9.86\times 10^{-6}$ S/cm of (sample Ⅰ) to 133.45 S/cm (sample Ⅳ) (Figure 3, top-right), as was evaluated by 4-point-measurements.

This shows that an increase of the methane/hydrogen gas flow ratio during growth leads to the reduction of the diamond film’s grain size and an increase in electrical conductivity, when the process pressure and filament current are kept the same.

**Figure 4:**
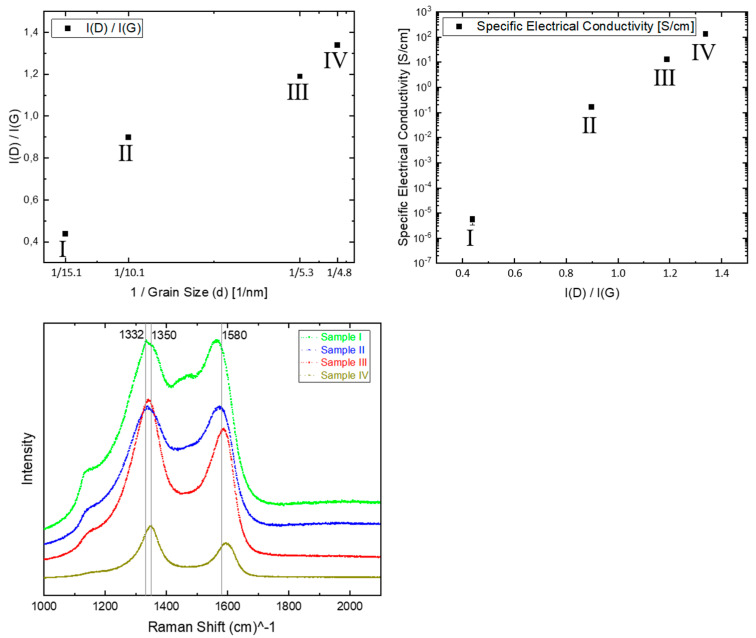
Evaluation of the correlation between the intensity ratio of D-peak and G-peak, namely I(D)/I(G), with diamond grain size (**top-left**) and with specific electrical conductivity (**top-right**), based on Raman-spectra (**bottom**) of four diamond film samples.

This is explained by an increase of grain boundary volume with a higher content of defective sp^2^ carbon sites that leads to the delocalization of π-electrons [13,22,23]. Insight into the chemical structure of the grain boundaries can be obtained by Raman measurements (Figure 4, bottom), especially the analysis of the peaks at 1332 cm^−1^ (bulk diamond), 1350 cm^−1^ (disordered graphitic band, D-peak), and 1580 cm^−1^ (graphite band, G-peak) [24,25]. An increasing content of defective sp^2^ carbon sites in the films can be deduced from a shift of the D- and G-peaks to higher peak position values, and more notably, the appearance of a D-peak, which is associated with disordered graphitic structures and the semi-metallic character of the electronic structure of graphite [25].

In sample Ⅰ, the bulk diamond peak at 1332 cm^−1^ can still be seen on top of the broad D-band peak. This shows a dominant fraction of sp^3^ bonds due to the relatively large crystalline nanodiamond grains. In contrast to that, sample Ⅳ shows only a broad intensive peak at 1350 cm^−1^, corresponding to a much smaller grain size and higher fraction of sp^2^ bonds, due to the higher grain boundary volume. Thus, the evaluation of the intensity of D-band I(D) is accomplished by an integrated fitting with separate fitting curves at 1332 cm^−1^ and 1350 cm^−1^. Figure 4 (top-left) demonstrates the correlation between the increasing intensity ratio of D-peak and G-peak, namely I(D)/I(G), with the inverse diamond grain size. Furthermore, this ratio was also assigned to evaluate the $L_{a}$ (in-plane correlation length within an ordered graphite layer) [25], and the degree of order of the clustered aromatic sp^2^ phase in graphitic materials [24], thus was found to accompany the higher specific electrical conductivity [12,26], as Figure 4 (top-right) shows.

**Figure 5:**
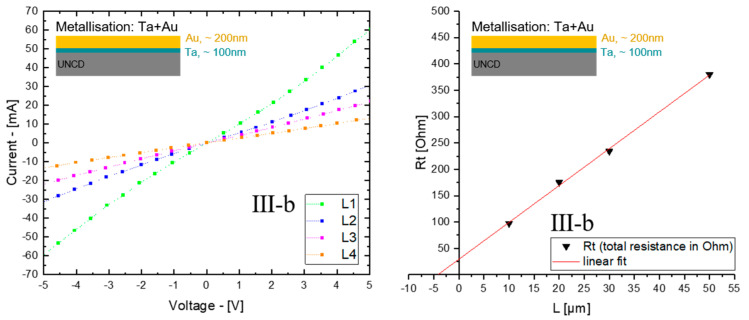
Ohmic I-V characteristic (**left**), and linear fitting of total resistance in Ohm (Rt) against LTLM pads distance L in µm (**right**), of subgroup sample Ⅲ-b, contact material with Ta/Au.

The measured contact resistance of samples subgroup Ⅲ-a to Ⅲ-c (based on LTLM and VDP micro-structures) provide the overall information that carbide-forming metals, as well as the single direct Au-contact, are suitable ohmic contact materials, since linear I-V characteristics were collected within a voltage from −5V to +5V (Figure 5, left, taking sample Ⅲ-b as an example). Because of the enhanced electrical conductivity of the diamond film, due to the reduced average grain size and the increased sp^2^ bonding fraction in the grain boundaries [13], this is distinctly different from the conventional doping process for a crystalline semiconductor, whose charge carriers are contributed by ionization of donor or acceptor. The contact between metallization materials and diamond films is therefore called a metal–semimetal contact [27].

**Figure 6:**
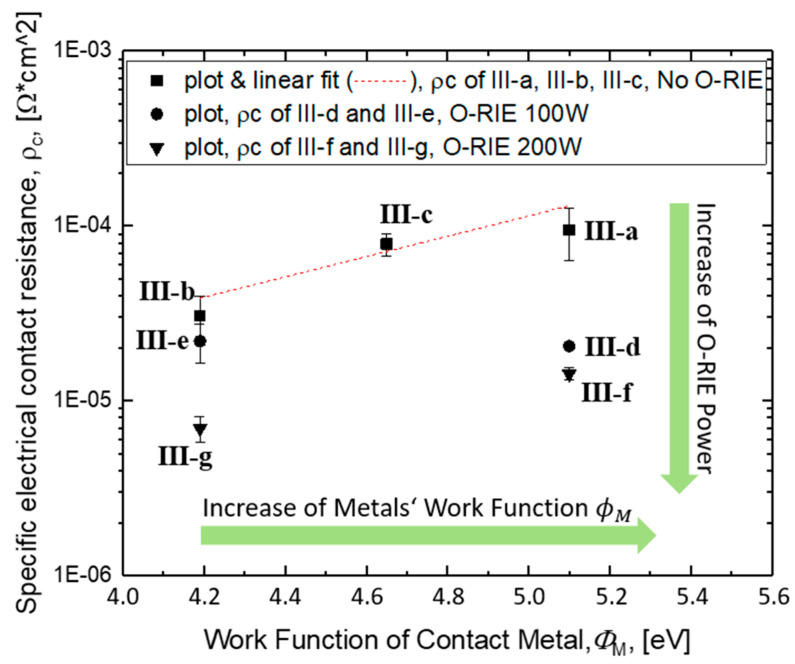
Specific electrical contact resistance (in ${\Omega \mathrm{cm}}^{2}$), regarding different contact metals’ work function and O-RIE process power.

Figure 5 (right) shows the exemplary analysis of the Ta/Au contact metallization. The linear fitting of total resistance (R_t_), which consists of R_sh_ (film resistance) and R_c_ (metal contact resistance), against the distance L among the four metallic pads (based on the LTLM micro-structure) is shown in Figure 5. The intercept of the fitted line with the x-axis refers to the transfer length $L_{t}$, which is the length under the metal contact, where the current in the film is reduced by 1/*e*, and the intercept with the y-axis reveals the contact resistance $R_{c}$. Table 2 gives an overview of the obtained contact resistivity for different metals. The results given in Table 2 indicate that Ta/Au as a combined metallization material shows a relatively small specific contact resistance among samples Ⅲ-a to Ⅲ-c, which also can be noted in Figure 6, after plotting the results regarding the three different measurement positions.

The samples’ surfaces of subgroup Ⅲ-a to Ⅲ-c were in the as-grown state, having an H-termination after HFCVD deposition in a hydrogen-rich atmosphere. We hence assume that the surface state and electron affinity (χ) of those three samples were the same. The high electron density of about $2.9\times 10^{19}\left({\mathrm{cm}}^{-3}\right)$ [23] observed in these kinds of films lets us assume that the density of surface states is not able to pin the fermi level at the surface. Thus, the barrier height (qϕ_Bn_) of a metal–semiconductor contact is determined entirely by the difference of the metal work function (ϕ_M_) and the electron affinity of the semiconductor (χ) [28]. The barrier height can be formulated as in [28]:

(1) $$q\phi_{\mathrm{Bn}}=q\left(\phi_{M\;}-\;\chi \right)-q\Delta \phi$$

where qΔϕ  is the image force barrier lowering. An increasing metal work function increases the specific contact resistance. In this experiment, the contact resistances on UNCD for Ta, Mo, and Au, whose $\phi_{M}$ are respectively ~ 4.19 eV (Ta), 4.36–4.95 eV (Mo), and 5.10–5.47 eV (Au) [29], are shown by square plots in Figure 6.

In the cases where an oxygen plasma-etching process was applied prior to the contact deposition, sample subgroups (Ⅲ-d to Ⅲ-g) indicated a drastically decreased contact resistance (circular and triangular plots in Figure 6, note the y-axis with logarithmic scaling). For Au as contact material, an obvious reduction was obtained, namely a decrease from $7.26\times 10^{-5}–1.30\times 10^{-4}$ ${\Omega \mathrm{cm}}^{2}$ (sample III-a) to $1.36\times 10^{-5}$–$1.56\times 10^{-5}$ ${\Omega \mathrm{cm}}^{2}$ (sample Ⅲ-f), with a factor of 5–8, which can only be attributed to the oxygen ion plasma treatment. Furthermore, an overall reduction by a factor of 12–16 can be recognized for Ta as a carbide-forming interlayer between Au and the diamond film (down to $6.03\times 10^{-6}$–$8.25\times 10^{-6}$ ${\Omega \mathrm{cm}}^{2}$ in sample Ⅲ-g).

However, an O-RIE process with different working powers resulted in quite different specific resistance reductions, whereby the samples Ⅲ-f and Ⅲ-g with higher power presented more significant changes. For a better understanding of the reason why the contact resistance decreased after those diamond film’s surfaces were pre-treated in oxygen ion plasma, especially under higher power oxygen plasma, further evaluation was completed with the aid of AFM, XPS, and surface wetting measurements.

**Figure 7:**
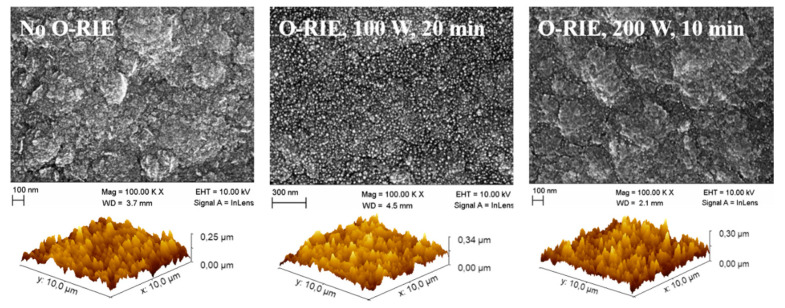
SEM and AFM measurements of sample surfaces, respectively, without O-RIE (**left**), with 100 W O-RIE (**middle**), and 200 W O-RIE (**right**) processes.

**Figure 8:**
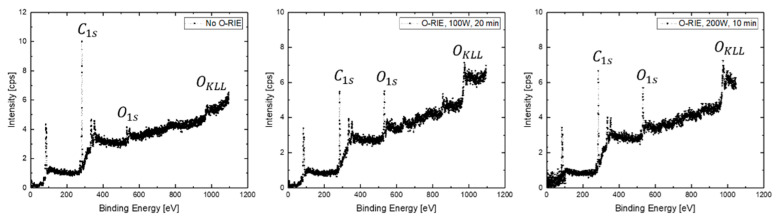
XPS survey scan spectra, respectively, for sample surfaces without O-RIE (**left**), with 100 W O-RIE (**middle**), and 200 W O-RIE (**right**) processes.

**Figure 9:**
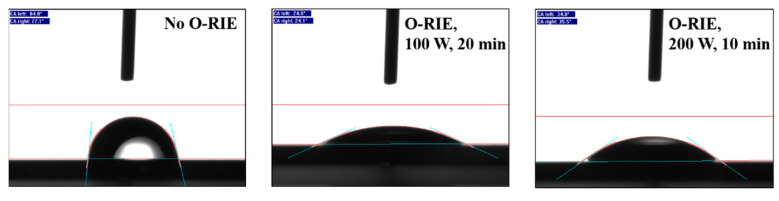
Contact angle measurements, respectively, for sample surfaces without O-RIE (**left**), with 100 W O-RIE (**middle**), and 200 W O-RIE (**right**) processes.

AFM gave information about the entire contact surface area, regarding the different surfaces’ roughness. The measurements were all performed on the same measurement size ($10\times 10{\;µm}^{2}$), as shown in Figure 7. However, the ratios between the area measured by the AFM and the resulting surface area did not show a large difference—respectively, 100/106 ${µm}^{2}/{µm}^{2}$ (sample without O-RIE), 100/108.5 ${µm}^{2}/{µm}^{2}$ (sample with 100 W O-RIE), and 100/108.9 ${µm}^{2}/{µm}^{2}$ (sample with 200 W O-RIE)—which means the surface roughness ($R_{\mathrm{rms}}$ respectively 32.2 nm, 40.8 nm, and 40.3 nm) is not the main reason for a significant reduction in the contact resistance on a logarithmic scale. A more plausible reason for the reduction in contact resistance is the different surface termination after O-RIE, which can be proved by different surface XPS spectra (Figure 8) and different water wetting angles on sample surfaces in macroscopy (Figure 9).

According to the XPS survey scan spectra, both of the samples that were etched with O-RIE processes (Figure 8, middle and right) exhibited a significant $O_{1s}$ intensity (~531 eV) and $O_{{\mathrm{KL}}_{23}L_{23}}$ intensity (~ 978 eV), whereas the sample without O-RIE (Figure 8, left) pre-treatment did not present a strong peak, even though a small peak was captured, probably due to some water adsorbed on the sample surface. The intensity ratio between the $O_{1s}$ peak (~531 eV) and the $C_{1s}$ peaks (~284.8 eV) of those three samples are respectively, 0.12, 0.65, and 0.52 (from left to right in Figure 8). This is also matching to the contact angle measurement results in Figure 9, which indicates a transfer from a hydrophobic surface to a hydrophilic surface, after an O-RIE process on diamond film, and furthermore an enhancement of surface energy according to Young’s equation [30]. Different plasma process durations for uniform remaining UNCD film thickness were also tested on identical diamond films, leading to comparable results of contact resistances to those presented earlier. However, the relative higher intensity ratio between the $O_{1s}$ peak and the $C_{1s}$ peaks of the sample pre-treated with lower oxygen plasma power (100 W) was caused by a longer O-RIE process duration. This can be explained by a higher level of surface defects, such as a combination of -CH_x_, =CH_x_ with C-O-O-C, -COH or C-O-V (vacancy) [31], but not clear surface reconstructions by the formation of ether-configuration (C-O) and ketone-configuration (C=O) oxygen-carbon atoms bonds. Those effective reconstructions contribute particularly sensitive to the diamond surface electron affinity change from the negative range (H-terminated) to the positive range (O-terminated) [32,33].

**Figure 10:**
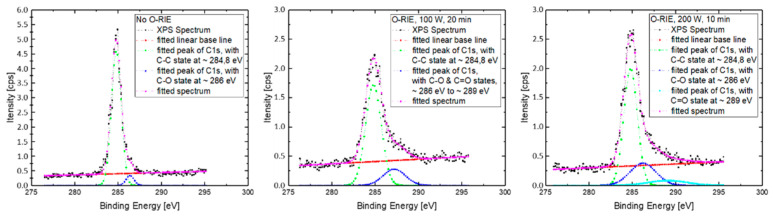
XPS batch scan spectra about $C_{1s}$ area, respectively, for sample surfaces without O-RIE (**left**), with 100 W O-RIE (**middle**), and 200 W O-RIE (**right**) processes.

A more detailed analysis of the $C_{1s}$ peaks indicates that the spectrum of sample surface pre-treated with 200 W power plasma (Figure 10, right) shows, first of all, the largest intensity ratio by 0.21, between $C_{1s}$ (C-O state, ~284.8 eV) and $C_{1s}$ (C-C state, ~286 eV), and furthermore, a clear splitting of the complete spectrum into three fitted sub-peaks, respectively, for $C_{1s}$ (C-C state, ~284.8 eV), $C_{1s}$ (C-O state, ~286 eV), and $C_{1s}$ (C=O state, ~289 eV) [34]. In comparison, the XPS spectrum of sample surface pre-treated with 100 W plasma (Figure 10, middle) did not present a clear fitting with sub-peaks of C-O state and C=O state inside the $C_{1s}$ area, but only a mixed wide peak ~287.2 eV for both of them; the XPS spectrum of sample surface without oxygen plasma pre-treatment (Figure 10, left) only indicates a weak intensity ratio of $C_{1s}$ in C-O state to $C_{1s}$ in C-C state by ~0.08 and no more captured intensity about the $C_{1s}$ in C=O state, which is metastable, compared with the C-O state, but with higher electron affinity [32,33]. Based on this, it is more clear to say that the samples’ surface pre-treated with the 200 W O-RIE process has an additional higher surface electron affinity (χ), as described in Equation (1), and thus shows further reduction of the total barrier height ($q\phi_{\mathrm{Bn}}$) between contact metals and the film material and therefore a more noticeable reduction in total contact resistance (Ⅲ-f and Ⅲ-g with triangular plots in Figure 6). In comparison to this, the samples’ surfaces pre-treated with lower plasma power did not show a clear forming of C-O or C=O state, which means no clear and stable surface state change; thus, in turn, there was no similar contact resistance reduction tendency in Figure 6 (Ⅲ-d and Ⅲ-e with circular plots). In short, the oxygen-terminated surfaces, especially the sample surface pre-treated with the maximal plasma power of 200 W, which leads to noticeable C-O and C=O bonding states, demonstrates a change of the surface electron affinity from the negative range (when hydrogen-terminated) to a positive range [33]. A consequence of an increase of sample surface electron affinity is a decrease of the Schottky barrier and the electrical contact resistance.

## 4. Conclusions

Controlled growth of non-conductive nanocrystalline diamond films (NCD) and high-conductive (ultra-)nanocrystalline diamond films (UNCD) was implemented only by varying the methane (${\mathrm{CH}}_{4}$) composition in the total precursor gas mixture. An increased methane gas flow leads to a decreasing grain size and an increase in grain boundary volume. Furthermore, an increased methane content during growth also increases the electrical conductivity of nanocrystalline diamond films, which is due to a higher amount of ordered sp^2^-bonding fractions in the grain boundary area. The smallest specific contact resistance that was achieved between metallization and conductive diamond film was $(6.03\times 10^{-6}$–$8.25\times 10^{-6}{)\;\Omega \mathrm{cm}}^{2}$, with Ta-Au as the metallization combination and the diamond surface pre-treated with the 200 W O-RIE process for short time. In comparison, the largest contribution to the reduction of contact resistance was made by the diamond film surface pre-treatment with O-RIE, which resulted in a change of the film surface state, namely the oxygen termination, and enhanced surface electron affinity. We showed that the contact resistance of a conductive nanocrystalline diamond film can be reduced by a factor of 16 by the right choice of contact material and by application of an O-RIE plasma etching step. This allows an improved sensitivity for sensors based on electrically conductive nanocrystalline diamond films, as well as an improved energy efficiency of nanocrystalline diamond actuators in microelectronic device structures.

## Figures and Tables

**Table 1:** Sample group based on different metallization and O-RIE parameters.

Sample	III-a	III-b	III-c	III-d	III-e	III-f	III-g
contact metal	Au	Ta	Mo	Au	Ta	Au	Ta
thickness	200 nm	100 nm	100 nm	200 nm	100 nm	200 nm	100 nm
cover metal	-	Au	Au	-	Au	-	Au
thickness		200 nm	200 nm		200 nm		200 nm
O-RIE, 100 W	No	No	No	Yes	Yes	No	No
O-RIE, 200 W	No	No	No	No	No	Yes	Yes

**Table 2:** Measurement results of specific electrical contact resistance and specific electrical conductivity of diamond film, based on different micro-structures and metallization metals.

Sample Subgroups	$\rho_{c},\;\Omega \times cm^{2}\;(\mathrm{LTLM})$	$\sigma_{f},\;S/cm\;(\mathrm{LTLM})$	$\sigma_{f},\;S/cm\;(\mathrm{VDP})$
Au (III-a)	$(9.47\pm 3.09)\times 10^{-5}$	10.67±0.72	11.56±0.67
Ta/Au (III-b)	$(3.07\pm 0.88)\times 10^{-5}$	12.79±0.49	13.81±0.39
Mo/Au (III-c)	$(7.89\pm 1.14)\times 10^{-5}$	11.29±0.22	11.75±0.04
Au (III-d)	$(2.05\pm 0.09)\times 10^{-5}$	8.77±0.09	9.08±0.06
Ta/Au (III-e)	$(2.19\pm 0.56)\times 10^{-5}$	8.14±0.11	8.38±0.02
Au (III-f)	$(1.43\pm 0.11)\times 10^{-5}$	9.49±0.02	10.01±0.02
Ta/Au (III-g)	$(6.98\pm 1.15)\times 10^{-6}$	9.16±0.07	9.59±0.05

## Data Availability

The data that support this research are available from the corresponding author upon reasonable request.
